# Supplementary material for: Identification and functional analysis of non-coding regulatory small RNA FenSr3 in Bacillus amyloliquefaciens LPB-18
Source: PeerJ. 2023 May 15;11:e15236. doi: 10.7717/peerj.15236 (PMC10194069; doi:10.7717/peerj.15236)
Supplement: Supplemental Information 4 [file peerj-11-15236-s004.zip › KO/CK-vs-T1_map/map05322.html]

KEGG PATHWAY: Systemic lupus erythematosus - Reference pathway


|  |  |
| --- | --- |
| **Systemic lupus erythematosus - Reference pathway** |  |

[
Pathway menu
| Organism menu
| Pathway entry
| Show description
| User data mapping
]

Systemic lupus erythematosus (SLE) is a prototypic autoimmune disease characterised by the production of IgG autoantibodies that are specific for self-antigens, such as DNA, nuclear proteins and certain cytoplasmic components, in association with a diverse array of clinical manifestations. The primary pathological findings in patients with SLE are those of inflammation, vasculitis, immune complex deposition, and vasculopathy. Immune complexes comprising autoantibody and self-antigen is deposited particulary in the renal glomeruli and mediate a systemic inflammatory response by activating complement or via Fc{gamma}R-mediated neutrophil and macrophage activation. Activation of complement (C5) leads to injury both through formation of the membrane attack complex (C5b-9) or by generation of the anaphylatoxin and cell activator C5a. Neutrophils and macrophages cause tissue injury by the release of oxidants and proteases.

|  |  |  |
| --- | --- | --- |
| Reference pathway | 184% 150% 122% 100% 82% 67% 55% | 图片下载 |
